# Phytochemical composition and in vitro antioxidant and antimicrobial activities of *Bersama abyssinica* F. seed extracts

**DOI:** 10.1038/s41598-024-56659-1

**Published:** 2024-03-15

**Authors:** Belayhun Alemu, Meseret Derbew Molla, Hiwot Tezera, Aman Dekebo, Tadesse Asmamaw

**Affiliations:** 1 Department of Biochemistry, School of Medicine, College of Medicine and Health Science, Dilla University, Dilla, Ethiopia; 2 Department of Biochemistry, School of Medicine, College of Medicine and Health Science, University of Gondar, Gondar, Ethiopia; 3 Flinders Health and Medical Research Institute, College of Medicine and Public Health, Flinders University, Adelaide, SA Australia; 4 Department of Applied Chemistry, School of Applied Natural Sciences, Adama Science and Technology University, Adama, Ethiopia

**Keywords:** Antimicrobial, Antioxidant, *Bersama abyssinica*, Extract, Phytochemical, Seeds, Biochemistry, Biological techniques, Biotechnology, Drug discovery, Biomarkers

## Abstract

Medicinal plants can be potential sources of therapeutic agents. Traditional healers use a medicinal plant from Ethiopia, *Bersama abyssinica* Fresen, to treat various diseases. This study aimed to investigate the phytochemical components and antioxidant and antimicrobial activities of *B. abyssinica* seed extracts (BASE). Gas chromatography coupled to mass spectroscopy (GC–MS) analysis was used to determine the phytochemical compositions of BASE. The antioxidant activities were assessed by using 2, 2-diphenyl-1-picrylhydrazyl (DPPH) assay, thiobarbituric acid-reactive species (TBARS) assay, ferric chloride reducing assay and hydroxyl scavenging capacity assay. Antimicrobial activity was investigated using the agar well diffusion method. Phytochemical screening showed the presence of saponins, glycosides, tannins, steroids, phenols, flavonoids, terpenoids, and alkaloids. GC–MS analysis revealed the presence of 30 volatile compounds; α-pinene (23.85%), eucalyptol (20.74%), β-pinene (5.75%), d-limonene (4.05%), and o-cymene (5.02%). DPPH-induced free radical scavenging (IC_50_ = 8.78), TBARS (IC_50_ = 0.55 µg/mL), and hydroxyl radicals’ scavenging capacities assays (IC_50_ = 329.23) demonstrated high antioxidant effects of BASE. Reducing power was determined based on Fe^3+^–Fe^2+^ transformation in the presence of extract. BASE was found to show promising antibacterial activity against *S. aureus*, *E. coli,* and *P. aeruginosa* (zone of inhibition 15.7 ± 2.5 mm, 16.0 ± 0.0 mm, and 16.7 ± 1.5 mm, respectively), but excellent antifungal activities against *C. albican* and *M. furfur* (zone of inhibition 22.0 ± 2.0 mm and 22.0 ± 4.0 mm, respectively). The **s**eeds of *B. abyssinica* grown in Ethiopia possess high antioxidant potential, promising antibacterial and superior antifungal activity. Therefore, seeds of *B. abyssinica* provide a potential source for drug discovery.

## Introduction

The flowering plants demonstrated medicinal value of which 30% of these plants constitute phytochemicals^1^. In Ethiopia, 60% of plants are supposed to be indigenous with their curative potential^2^. A medicinal plant from Ethiopia, namely *Bersama abyssinica* Fresen, has been used by the traditional healer to cure many diseases^3^. *B. abyssinica* is a species of medium-sized evergreen shrub and tree that is distributed across sub-Saharan Africa^4^. Traditionally, all parts of the plants have been used for the treatment of many diseases. The Zulu community uses the leaves of *B. abyssinica* to cure barrenness, impotence, menstrual pain, and leprosy and to protect charm^5^. In Kenya, boiled roots and leaves are used for the treatment of sexually transmitted diseases, stomachache, pneumonia, TB, and malaria^6^.

In Ethiopia, *B. abyssinica* has various treatment purposes traditionally. Crushed fresh root mixed with cold water is taken orally for treatment of bronchitis and febrile illness^7^. The fruit and root powder is mixed with honey or butter and applied to cure wound and eczema^8^. Leaves and bark of roots are employed for the treatment of hypertension, rabies, cough, ascaris, diarrhea, rheumatoid, and wound^9^. A liquid preparation of buds has been reported as a remedy against dysentery and roundworms, while an infusion prepared from the stem bark is orally taken to treat cancer-like symptoms^10^. The leaf is boiled and drunk to cure diarrheal diseases^11^. The Ari and Male communities use the bark and buds for snake bite and liver disease treatment^12^. Sidama people use the bark by powdering, dissolving, boiling, and drinking small amounts for the treatment of tumors^13^. Gurage and Silti communities use the root, stem, leaf, and seed of *B. abyssinica* for curing lung disorders, heart failure, back pain, gonorrhea, intestinal problems, skin diseases, and abnormal menstrual cycles^14^.

The pathogenesis and prognosis of diseases such as cancer^15^, wound^16^, diabetes^17^ and malaria^18^ are associated with oxidative stress which traditional healers treat these diseases with extracts from *B. abyssinica*. Antioxidant therapy may have a positive impact on the course of many diseases, and some available synthetic antioxidants have been reported to cause adverse health-related issues, such as cancer^19^. Additionally, the shortage of antimicrobial agents is raised from time to time due to emerging and re-emerging infections, emergency drug resistance pathogens, adverse effects, and the high cost of synthetic drug development^20,21^. Hence, there is a need to search for new antioxidant and antimicrobial agents with better effects, less toxicity, and affordable cost, of which compounds originating from natural medicinal plants are alternatively recommended^22^.

To the best of our knowledge, analysis of phytochemical composition, and biological activities of seeds extract of *B. abyssinica* had not been reported so far. This study aimed to investigate the phytochemical composition and antioxidant and antimicrobial properties of the *B. abyssinica* seed extract (BASE).

## Materials and methods

### Plant material collection and identification

The seeds of *B. abyssinica* were collected from Semar village, Sude district in Ethiopia at the latitude of 7° 55′ 48′′ N, longitude of 39° 55′ 12′′ E, and altitude of 2549 m above sea level^23^. The seeds were gifted from Getaw Alemu who is the owner of the land. The sample was pressed, identified, and authenticated at the Department of Biology, University of Gondar (UoG) by the Botanist Getnet Chekole, and a voucher specimen (01/BA/2022) was deposited in the UoG Herbarium.

### Extraction of the seeds of *B. abyssinica*

The seeds of *B. abyssinica* were washed with tap water, rinsed with distilled water, and air-dried at room temperature for two weeks under shed^24^. The seeds were grounded using a mechanical grinder machine (British Cult, Ser.no 15-303/1066). The pounder of the seeds (450 g) of *B. abyssinica* was soaked in 80% methanol for 72 h and occasional shaking. The procedure was repeated three times. Then, each extract was filtered by Whatman No. 1 filter paper twice and concentrated using a rotatory evaporator (YAMATO CF 301, Japan). The concentrated extract was incubated at 40 °C in an oven for 3 days and placed in a deep freezer at − 140 °C overnight to be lyophilized (LABFREEZ FD-12-MR Vacuum Freeze Dryer, China). Powder of dried seeds of *B. abyssinica* (100 g) was hydro-distilled for 4 h by using a Clevenger apparatus to isolate the volatile compounds.

### Phytochemical screening test

*Test for saponin* 0.5 g of the plant extract mixed in 10 mL distilled water. The suspension shook in a test tube for 5 min. The formation of the foam indicates the presence of saponin^25^. *Test for flavonoid* 1 mL plant extract was added into 2 mL of 2% NaOH solution with a few drops of dilute HCl. An intense yellow color change to colorless indicates flavonoids^26^. *Test for terpenoids* Adding 2 mL chloroform in 5 mL plant extract followed by the addition of 3 mL concentrated H_2_SO_4_ and boiling in a water bath would result in a grey-colored solution that shows the presence of terpenoid^26^. *Test for phenols* 2 mL of plant extract was treated with 5% FCl_3_. The formation of blue or black color indicated the presence of phenols^27^. *Test for tannins* 0.1% FeCl_3_ was added to crude extracts and the formation of a brownish-green or a blue-black color shows the presence of tannins^27^. *Test for glycosides* 0.5 mL glacial acetic acid and 2–3 drops of FeCl_3_ were mixed with 2 mL of extract. Then, add 1 mL of 98% H_2_SO_4_ along the walls of the test tube. A deep blue color was formed at the junction of two liquids showing the presence of glycosides^28^. *Test for steroids* A red color was produced at the lower layer when 2 mL of extract was dissolved in 2 mL chloroform and 2 mL of 98% H_2_SO_4_ shows the presence of steroids^29^. *Test for alkaloids* 0.5 mg of crude extract with 1% HCl was stirred in a water bath for 5 min and was filtered into to test tube. Up on the addition of 1 mL Wagner’s reagent, the formation of a brown/reddish precipitate indicates the presence of alkaloids^30^.

### Gas chromatography–mass spectrometry analysis

GC–MS analysis of BASE was performed on GC coupled to MS (Mass Hunter GC/MS Version B.07.03.2129, Agilent Technologies, USA). The carrier gas was helium flowing at a rate of 1 mL/min. Aliquots of hydro-distillate (1 mL of 1 ppm in hexane) were injected. The injector temperature was 230 ℃. The temperature of the oven was begun at 40 ℃ hold for 5 min and then, it was raised to 250 ℃ hold for 20 min for a total run-time of 60 min. Mass spectra were recorded at 70 eV, scanning the 50–500 m/z range which was connected to a computer Mass Spectra data bank. The components were identified by comparing their retention times with the retention times of authentic standards, and mass spectra with the National Institute of Standards and Technology (NIST 2017). The GC–MS was done in the JIJE LABOGLASS chemistry analysis laboratory, in Addis Ababa, Ethiopia.

### In vitro anti-oxidant activity

The antioxidant activity of BASE was evaluated using 2, 2-diphenyl-1-picrylhydrazyl (DPPH) radical scavenging assay^31^, thiobarbituric acid-reactive species (TBARS) assay^32^, ferric chloride reducing assay^33^, and hydroxyl radicals’ scavenging capacities^34^ that are described in the literature with slight modifications. All experiments were performed in triplicate.

In the DPPH radical scavenging assay, the extract was prepared in test tubes containing methanol to give 1000, 500, 250, 125, and 62.5 µg/mL solution. About 4 mL of 0.1 mM methanolic solution of DPPH was dropped into 1 mL solution from each test tube. Ascorbic acid was used as a positive control and was prepared as above without extract. The resulting mixture was placed in an oven at 37 °C for 30 min and subjected to a UV Visible Spectrophotometer (CARY 60, Malaysia) to record absorbance at 517 nm. The blank solution was prepared without a sample or ascorbic acid. The percentage of DPPH Free radical scavenging (FRS) was manipulated by the formula.

$${\text{FRS}}=\left(\frac{{\text{AB}}-{\text{AS}}}{AB}\right)\times 100,$$

where AB and AS are the absorbance values of the blank and test samples, respectively. Free radical scavenging activity was measured as the IC50 value which was defined as the concentration required for 50% inhibition, as compared to the control.

In the TBARS assay, egg homogenate (250 μL, 10% in distilled water, v/v) and 50 μL of extract were mixed in a test tube and the volume was made up to 500 μL, by adding distilled water. Then, 25 μL of 0.07 M ferrous sulfate (FeSO4) was added to the above mixture and incubated for 30 min, to induce lipid peroxidation. Thereafter, 750 μL of 20% acetic acid (pH 3.5) and 750 μL of 0.8% thiobarbituric acid (TBA) (w/v) (prepared in 1.1% sodium dodecyl sulfate) and 25 μL 20% trichloroacetic acid (TCA) was added, vortexed, and then heated in a boiling water bath for 60 min. After cooling, 3 mL of 1-butanol was added to each tube and centrifuged at 3000 revolutions per minute for 10 min. The absorbance of the organic upper layer was measured at 532 nm. For the blank solution, 50 μL of distilled water was used in place of the extract or standard control. Ascorbic acid was used as the positive control and the percent of lipid peroxidation inhibition (I) was calculated by the formula:

$${\text{LPI}}=\left(\frac{{\text{AB}}-{\text{AS}}}{AB}\right)\times 100,$$

where AS is the value absorbance of the test sample and AB is the absorbance value of the blank.

In the ferric chloride reducing assay, 2.5 mL (1 mg/mL) of extract was mixed with 2.5 mL sodium phosphate buffer (pH 6.6, 0.2 mol/L) and 2.5 mL potassium ferricyanide (1%). The reaction mixture was incubated for 20 min at 50 °C. Then, 2.5 mL trichloroacetic acid (10%) was added and the mixture was centrifuged at 650 revolutions per minute for 10 min. After centrifugation, 5 mL supernatant was mixed with 5 mL distilled water and 1 mL of 0.1% ferric chloride. The absorbance was recorded at 700 nm. The absorbance of the reaction mixture would be increased with concentration which indicated reducing power. Ascorbic acid was used as standard.

In hydroxyl radicals’ scavenging capacities, first, 10.40 g of disodium hydrogen phosphate dodecahydrate (Na_2_HPO_4_·12H_2_O) and 1.32 g sodium dihydrogen phosphate dihydrate (NaH_2_PO_4_·2H_2_O) were measured and dissolved in 250 mL distilled water to the phosphate buffer solution. Then, 13.9 mg Ferrous sulfate heptahydrate (FeSO_4_·7H_2_O) and 37.23 mg ethylenediamine tetraacetic acid (EDTA) salt were added to distilled water and were made up to a constant volume of 100 mL to obtain the EDTA–Fe^2+^ solution. Next, 9 mg safranin N was dissolved in the previously prepared phosphate buffer solution to a constant volume of 100 mL. All of the above solutions were fresh and photophobic. Finally, 1 mL of extract or standard control, 0.5 mL of EDTA–Fe^2+^ solution, 1 mL of potassium phosphate buffer solution, 1 mL of safranin N solution, and 1 mL of 3% hydrogen peroxide (H_2_O_2_) solution were added to the order in a test tube. The mixture was incubated for 30 min at 37 °C. The ultraviolet spectroscopy absorption was measured at 520 nm. Ascorbic acid was used as standard. The percentage of hydroxyl radicals’ scavenging capacities (PHRSC) was calculated using the formula

$${\text{PHRSC}}=\left(\frac{{\text{AB}}-{\text{AS}}}{AB}\right)\times 100,$$

where AS is the absorbance value of the sample and AB is the absorbance value of the blank.

### Antimicrobial activity test

The antimicrobial activities were investigated against bacteria and fungi. Bacteria include: *Staphylococcus aureus* American Type Culture Collection (ATCC43502), *Pseudomonas aeruginosa* ATCC2785 and *Escherichia coli* ATCC25922. Fungi include *Candida albicans* and *Malassezia furfur*. The bacteria strains were obtained from the microbiology laboratory of the National Clinical Bacteriology and Mycology Reference Laboratory of the Ethiopian Public Health Institution (EPHI), and fungi were clinically isolated from UoG Teaching Hospital. The test organisms were kept on tryptic soy broth supplemented with 20% glycerol at − 80 °C in the microbiology laboratory of UoG.

Inoculum preparation was according to recommended by the Clinical and Laboratory Standards Institute^35^. All test organisms were grown in Petri dishes containing agar medium specific to each microorganism as a refreshment of each strain for the actual test. Each bacterial strain was incubated for 24 h at 37 °C and each fungus was incubated for 7 days at 25 °C. Standardization was by taking 3–5 inoculums from a fresh, pure culture of the test organism and making a suspension with nutrient broth for bacteria and sabouraud dextrose broth for fungi. Then, these suspensions were diluted with appropriate broth in 1:10 to get 1 × 10^7^ CFU/mL and 1 × 10^6^ spore/mL bacteria and fungi, respectively.

An antimicrobial activity of the plant extracts was conducted using the agar well diffusion method^36,37^. A sterile cotton swab was dipped into the adjusted suspension and the bacterial inoculum was uniformly spread on a sterile Petri dish Mueller–Hinton agar (the culture media was sterilized for 15 min at 121 °C). This procedure was repeated by streaking two more times, rotating the plate approximately 60° each time to ensure an even distribution of inoculums. The rim of the agar was swabbed. The extracts were diluted in 5% dimethyl sulfoxide (DMSO) to yield concentrations of 50 and 20 mg/mL solution for bacteria and fungi tests respectively. DMSO is used because it cannot inhibit the growth of test organisms at this concentration. 120 μL volume of extracts, positive control, and negative control were added to each of the 3 wells (6 mm diameter holes cut in the agar gel, 20 mm apart from one another). The plates were incubated face up for 24 h at 37 °C under aerobic conditions. A zone of inhibition created around wells was used as an indicator of antimicrobial activities. Ceftriaxone and ketoconazole were positive controls used for bacteria and fungi respectively whereas well-filed with DMSO was negative control^38^. All tests were done in triplicate.

### Data analysis

The antimicrobial inhibition diameter and antioxidant inhibition percentages were reported as the mean ± standard deviation of the three consecutive tests. The statistical differences in the antimicrobial activity of extracts on each microorganism were carried out by employing one-way analysis of variance (ANOVA) followed by Post hoc Tukey’s tests. The IC_50_ value (50% inhibition) was calculated using regression equations obtained from the graph between percentage (%) inhibition and concentration. The criterion for significance was set at p < 0.05.

### Ethical clearance

The research was conducted in accordance with ethical review committee of UoG. Ethical clearance was obtained from Institutional Review Committee School of Medicine, College of Medicine and Health Science, UoG. Plant material collection and use is carried out in accordance with all relevant guidelines to International Convention on the Trade in Endangered Species of Wild Fauna and Flora.

## Results

### Phytochemical screening

The phytochemical screening test of *Bersama abyssinica* seed extract (BASE) showed the presence of various phytochemicals (Table 1).

**Table 1:** Phytochemical screening test result.

No.	Phytochemical	Reagent	Extract
1	Alkaloids	Wagner’s	+
2	Glycosides	Keller Killian’s	+
3	Tannins	Foam test	+
4	Steroids	Ferric chloride	+
5	Phenols	Ferric chloride	+
6	Flavonoids	Shinoda test	+
7	Saponins	Foam test	+
8	Terpenoids	Salkowski test	+

+Presence of phytochemical.

### GC–MS analysis

GC–MS analysis led to the identification of 30 volatile compounds from the seed extract of *B. abyssinica*, representing 100% of the total peak area. Most of the identified compounds belonged to terpene hydrocarbons (65%) which include α-pinene (23.85%), eucalyptol (20.74%), β-pinene (5.75%), and d-limonene (4.05%), aromatic hydrocarbon such as o-Cymene (5.02) and other 2,4-Decadienal, (E, E) (5.50%), Furan, 2-pentyl- (4.81) and Nonanal (4.66) were found to be the main components (Table 2).

**Table 2:** The composition of the volatile compounds of BASE by GC–MS analysis.

S/N	Compound name	RT	Peak area	%Peak area
2	α-Pinene	8.66	6,186,960	23.85
3	Camphene	9.30	94,979	0.37
4	Cyclohexane, 1-methylene-4-(1methylethenyl)-	10.29	316,768	1.22
5	(−)-β-Pinene	10.46	1,490,872	5.75
6	α-Terpinene	12.24	260,800	1.01
7	d-Limonene	12.84	1,053,208	4.06
8	(1S)-2,6,6-Trimethylebicyclo [3.1.1] hept-2-ene	13.03	140,262	0.54
9	(1R)-2,6,6-Trimethylebicyclo [3.1.1] hept-2-ene	13.87	55,922	0.22
10	Gamma-terpinene	14.45	755,775	2.91
11	Tetradecane,2,6,10-trimethyl-	23.27	68,812	0.27
12	Eucalyptol	13.03	5,379,219	20.74
13	Linalool	16.80	32,718	0.13
14	Bicyclo [2.2.1] heptane-2-one-1,7,7-trimethyl-, (1S)-	17.62	55,155	0.21
15	Camphor	18.29	307,105	1.18
16	p-Cymene-2-ol methyl ether	20.35	47,106	0.18
**17**	2-Decenal, (E)-	21.06	904,231	3.49
18	2,4-Decadienal, (E, E)-	22.09	1,426,167	5.50
19	2-Undecenal	22.81	747,726	2.88
20	Nonanal	16.95	1,209,118	4.66
21	Decanal	19.88	430,857	1.66
22	o-Cymene	12.63	1,301,606	5.02
23	Toluene	3.78	646,645	2.49
24	Benzene, pentyl-	18.55	259,691	1.00
25	Nonane	7.42	51,937	0.02
26	Undecane	16.69	93,413	0.36
27	Trans-2-Decen-1-ol, methyl ether	11.53	71,856	0.28
28	Furan,2-pentyl-	11.12	1,248,678	4.81
29	3-trans-(1,1-dimethyl ethyl)-4-trans-meth-oxy cyclohexanol	19.67	89,940	0.35
30	Cyclohexasiloxane, dodecamethyl-	21.68	757,034	2.92

Compounds are listed in order of their elution.

### In vitro antioxidant activity

DPPH radical scavenging activity of BASE was assessed and compared with the standard ascorbic acid. The sample extract exhibited strong free radical scavenging activities compared to the standard. The percent of free radical inhibition was increased in a concentration-dependent (Table 3).

**Table 3:** Percentage of DPPH free radical scavenging activity and hydroxyl radical scavenging capacity.

Concentration (µg/mL)	DPPH percentage inhibition (mean ± SD)		Hydroxyl radicals’ scavenging capacities (mean ± SD)	
	BASE	Ascorbic acid	BASE	Ascorbic acid
62.5	85.14 ± 1.16*	40.07 ± 0.01*	16.43 ± 1.29**	31.35 ± 0.07**
125	87.45 ± 0.03*	57.46 ± 0.20*	25.39 ± 0.82**	43.77 ± 0.032**
250	89.76 ± 0.03*a	89.58 ± 0.03*	42.74 ± 1.43**	56.22 ± 0.03**
500	90.81 + 0.05*a	91.26 ± 0.00*	59.13 ± 0.36**	74.92 ± 0.05**
1000	93.39 ± 0.04*	94.91 ± 0.00*	75.97 ± 0.20**	83.06 ± 0.03**
IC50	8.78	75.44	329.23	166.75

Each value is mean ± SD (n = 3).

Values with * are significantly different between the groups at (P < 0.05). Values with the letter “a” do not show significant differences from each other. The values with ** are significantly different at p < 0.05.

**Figure 1:**
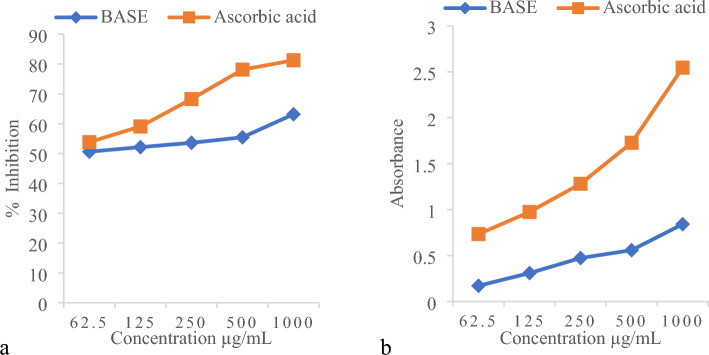
Antioxidant activity of BASE (**a**) percentage (%) lipid peroxidation inhibition induced by ferrous sulfate BASE IC_50_ = 0.55 µg/mL, Ascorbic acid IC_50_ = 1.10 µg/mL and (**b**) absorption of ferric chloride reducing assay, results were reported as mean ± SD of the three experiments.

Inhibition of lipid peroxidation was assessed by TBARS assay. BASE showed lipid peroxidation inhibition in a concentration-dependent (Fig. 1a).

Ferric ion-reducing power was assessed in the ferric chloride-reducing assay. The color change from yellow in the blank sample to different strengths of green in the extract was observed which indicated BASE has reduced capacity of the ferric ion. The reducing power of extracts increased in a concentration-dependent (Fig. 1b).

The hydroxyl radicals’ scavenging capacity BASE was assessed. BASE demonstrated moderate hydroxyl radicals’ scavenging activity. The percentage of hydroxyl radicals’ scavenging capacity was increased in a concentration-dependent (Table 3).

### Antimicrobial activity test

The extract showed antimicrobial activity against selected pathogenic bacteria and fungi at a concentration of 50 mg/mL and 20 mg/ mL, respectively. The results are presented in Table 4.

**Table 4:** Inhibition zone diameter (mm) of BASE against selected bacteria and fungi strains.

Sample	Concentration mg/mL	The zone of inhibition diameter (mm), including well diameter (6 mm)				
		*S. aureus*	*E. coli*	*P. aeruginosa*	*C. albican*	*Malassezia furfur*
BASE	50	15.7 ± 2.5*	16.0 ± 0.0*	16.7 ± 1.5*		
BASE	20				24.7 ± 1.5*	22.0 ± 4.4*
CE	50	33.3 ± 2.5*	31.1 ± 2.1*	27.3 ± 2.1*		
Keto	20				22.0 ± 2.0*	22.0 ± 4.0*
DMSO	50	6.0 ± 0.0	6.0 ± 0.0	6.0 ± 0.0		
DMSO	20				6.0 ± 0.0	6.0 ± 0.0

Values are expressed as Mean ± SD (n = 3), 6.0 ± 0.0 = no inhibition).

*CE* ceftriaxone, *Keto* ketoconazole, *DMSO* dimethyl sulfoxide.

Means * are significantly different between the group at p < 0.05.

## Discussion

The phytochemical screening determined the presence of alkaloids, glycosides, tannins, steroids, phenols, flavonoids, saponins, and Terpenoids which may be attributed to the traditional use of the plant as medicine. This finding is in line with the study on the root of *B. abyssinica* methanolic extracts^39^. There is a slight difference with a study on stem bark and leaves of *B. abyssinica* extracts^4,40^. A study on *Bersama engleriana* methanolic leaf extract of the phytochemical screening showed some variation in phytochemical content^41^. The difference might be due to the differences in genetic factors, oncogenic factors, morphogenetic factors, environmental factors, adaptation to local conditions, interactions with other organisms, and evolutionary history^42,43^.

GC–MS analysis revealed the presence of 30 volatile compounds. Most of the identified volatile compounds belonged to terpenes (64.38%) among which α-pinene (23.85%), eucalyptol (20.74%), β-β-pinene (5.75%), and d-limonene (4.05%) were found to be the main components. The results obtained from the seed extract were relatively different from the root, stem, and leaf of *B. abyssinica* GC–MS analysis^44^. A study done on a leaf of *B. abyssinica* extracts of the active fraction of GC–MS analysis also provided different phyto-constituents from this study^45^. Phytochemicals are found in different quantities in the cells, tissues, and organs of various plants. Since phytochemicals are complex and diverse in different parts of medicinal plants, the different secondary metabolites may be synthesized through special regulatory pathways and special transport routes in certain parts of the plants^43^. The specific tissues as well as the developmental stages, impact the pattern of gene expression related to phytochemical biosynthesis^42^. The α-pinene, eucalyptol, and β-pinene showed antimicrobial, antioxidant, and anti-inflammatory activities^46,47^. d-limonene, o-cymene, nonanal, furan,2-pentyl,2,4- and decadienal, (E, E) which have been reported by other studies with activities of antioxidants, antimicrobial, antifungal, and anti-inflammatory that was also existed in seeds of *B. abyssinica*^48–50^.

Multiple antioxidant assays were conducted to evaluate the antioxidant properties of BASE: DPPH radical scavenging assay, TBARS assay, OH radicals’ scavenging capacities assay, and ferric chloride assay on *B. abyssinica* seeds extract. Antioxidants are used to scavenge free radicals, prevent lipid peroxidation, and inhibit free-radical damage to biological systems so that they protect the body from oxidative stress. Nowadays, natural antioxidants from plants are recommended as preventive and treatments for many diseases^51^.

DPPH-induced free radical scavenging activity has been anticipated to be the principal method for determining the antioxidant properties of extracts^52^. This study results for the DPPH radical scavenging assay showed IC_50_ values of 8.77 µg/mL which is strong free radical scavenging activities compared to the ascorbic acid (IC_50_ = 75.44 µg/mL). Our study results coincide with the previous studies on roots, stem bark, and leaves of *B. abyssinica*. A previous study found the DPPH scavenging potential of solvent extracts of stem bark *B. abyssinica* with marked activity being exhibited by the methanol extract, followed by the water extract and ethyl acetate extract^4^. In another study, leaves of *B. abyssinica* showed the scavenging potential to scavenge DPPH free radicals^53^. The DPPH scavenging activity of *B. abyssinica* leaves and twigs has been reported^54^. In early studies, the root, stem, and leaves of *B. abyssinica* are rich in polyphenols, flavonoids, terpenes, alkaloids, and saponins compounds^4,44,53^ that the extracts possess hydrogen donating and radical scavenging ability.

Lipid peroxidation is a free radical-induced oxidative chain reaction by which one lipid molecule oxidation initiates the next lipid molecule up to the maximum possible amount forming lipid peroxide^55^. Ferrous ion was a potent initiator of lipid peroxidation^56^. Lipid peroxidation is terminated when the amount of ferrous iron oxidized is limited by the peroxyl radical reacted with antioxidants^57^. The absorbance values obtained by the TBARS method show the whole peroxide values formed by the oxidation of polyunsaturated acid^58^. The incubation of the egg homogenate in the presence of Fe^2+^ caused a significant increase in the malonaldehyde (MDA)^59^. This study result showed that BASE decreased MDA production. The IC_50_ values of the extract (0.55 µg/mL), and ascorbic acid (1.10 µg/mL) were shown to have a strong inhibitory effect significantly (p < 0.05) on Fe^2+^ induced lipid peroxidation in egg-yolk homogenates. The decrease in the Fe^2+^-induced lipid peroxidation in the egg-yolk homogenates in the presence of the extract could be due to the capacity of the extracts to scavenge free radicals and/or chelate ferrous ions.

In the ferric chloride assay, A reducing power assay was used to measure the free radical scavenging antioxidant activities of the crude extracts. The reducing agents in the sample reduce the ferric ion to the ferrous form. Higher reducing agents’ concentration means a large amount of ferrous ions^60^. Reduction of ferric ions and/or iron chelation would cause a decrease in free radicals formation^61^. In this study, the iron-reducing capacity of BASE was estimated from its ability to reduce the ferricyanide (Fe^3+^) to the ferrous (Fe^2+^) form by donating an electron. The extract showed a good reducing power capacity, which was concentration-dependent. This study’s results are consistent with the data published previously on methanolic leaf extract of the *B. abyssinica*. The antioxidant activity and reducing power capacity of the extracts were likely due to the presence of polyphenols and flavonoids, which can act as free radicals scavengers by donating hydrogen or electrons.

Hydroxyl radicals are the most harmful reactive oxygen species which are major contributors to oxidative damage in many biological systems^62^. In vitro, assays for the hydroxyl radical scavenging capacity are usually based on the scavenging activity of hydroxyl radicals. The extract showed good hydroxyl radical scavenging activity. The polyphenolic compounds especially terpenoids and flavonoids ability to quench hydroxyl radicals might directly relate to the prevention of lipid peroxidation^63,64^.

Because of the rise in antimicrobial-resistant microorganisms, the use of compounds extracted from medicinal plants may be helpful in the development of antimicrobial agents^65^. This study result showed promising antibacterial activities of the BASE against *S. aureus*, *E. coli*, and *P. aeruginosa*, with a zone of inhibition 15.7 ± 2.5 mm, 16.0 ± 0.0 mm, and 16.7 ± 1.5 mm, respectively. The positive standard, ceftriaxone, gave the zones of inhibition for *S. aureus* (33.3 ± 2.5 mm), *E. coli* (31.1 ± 2.1 mm), and *P. aeruginosa* (28.8 ± 0.2 mm) *w*ere in line with the published zones of inhibitions; 25–31 mm, 29–35 mm, and 18–22 mm, respectively^66^. The extract exhibited good antibacterial activities against *S. aureus* and *E. coli* when compared with a previous study^39^. Further comparison with literature reported for the stem bark extract that reported zone inhibition diameter of 15 mm and 10 mm *S. aureus* and *P. aeruginosa* respectively^67^. However, this study’s results show better antimicrobial activities compared to the methanolic stem bark extracts of *B. abyssinica.* The study found better antibacterial activity at lower concentrations against similar bacterial strains compared with the root, stem, and leaf extract of the same plant.

BASE showed zones of inhibition of 24.7 ± 1.5 mm and 22.0 ± 4.4 mm against *C. albican* and *Malassezia* spp., respectively whereas ketoconazole gave zones of inhibitions *C. albican* (22.0 ± 2.0 mm) and *M. furfur* (22.0 ± 4.0 mm) which is consistence with other studies^68,69^. Methanolic stem extract of *B. abyssinica* showed a 5.5 mm zone of inhibition for *C. albican*^67^ whereas this study results showed 24.7 ± 1.5 mm for *C. albican*. The difference in antimicrobial activities with other studies might be possibly due to the difference in concentration and types of antimicrobial agents in different parts of the study plant, environmental factors, and the laboratory method.

## Conclusion

In this study, the seeds of *B. abyssinica* were macerated and hydro-distilled to explore its biological activities. The seeds of *B. abyssinica* showed better antioxidant activities; however, they demonstrated good antimicrobial potential against *S. aureus, P. aeruginosa,* and *E. coli* and superior antifungal activities against *C. albicans* and *Malassezia* spp. Overall, the results of the study suggest that the composition of seeds of *B. abyssinica* is responsible for its strong antioxidant and antimicrobial properties. Therefore, seeds of *B. abyssinica* will be a potential source for the discovery of drugs. However, the compounds responsible for the antioxidative and antimicrobial activity are currently unclear. Therefore, further investigation is needed to isolate, identify, and characterize the antioxidant and antimicrobial compounds present in the seeds of the study plant.

## Data Availability

The data used to support the findings of the present study are available from the corresponding author.

## Abbreviations

ATCC: American type culture collection
BASE: *Bersama abyssinica* seed extract
CFU: Colony forming unity
DMSO: Dimethyl sulfoxide
DPPH: Diphenyl picrylhydrazyl
GC–MS: Gas chromatography–mass spectroscopy
FRS: Free radical scavenging
HRSC: Hydroxyl radicals’ scavenging capacities
IC_50_: Half inhibition concentration
MDA: Malondialdehyde
NIST: National Institute of Technology and Standards
TBARS: Thiobarbituric acid reactive species
